# Multi-channel MRI segmentation of eye structures and tumors using patient-specific features

**DOI:** 10.1371/journal.pone.0173900

**Published:** 2017-03-28

**Authors:** Carlos Ciller, Sandro De Zanet, Konstantinos Kamnitsas, Philippe Maeder, Ben Glocker, Francis L. Munier, Daniel Rueckert, Jean-Philippe Thiran, Meritxell Bach Cuadra, Raphael Sznitman

**Affiliations:** 1 Radiology Department, CIBM, Lausanne University and University Hospital, Lausanne, Switzerland; 2 Ophthalmic Technology Group, ARTORG Center Univ. of Bern, Bern, Switzerland; 3 Biomedical Image Analysis Group, Imperial College London, London, United Kingdom; 4 Unit of Pediatric Ocular Oncology, Jules Gonin Eye Hospital, Lausanne, Switzerland; 5 Signal Processing Laboratory, Ećole Polytechnique Fédérale de Lausanne, Lausanne, Switzerland; Bascom Palmer Eye Institute, UNITED STATES

## Abstract

Retinoblastoma and uveal melanoma are fast spreading eye tumors usually diagnosed by using 2D Fundus Image Photography (Fundus) and 2D Ultrasound (US). Diagnosis and treatment planning of such diseases often require additional complementary imaging to confirm the tumor extend via 3D Magnetic Resonance Imaging (MRI). In this context, having automatic segmentations to estimate the size and the distribution of the pathological tissue would be advantageous towards tumor characterization. Until now, the alternative has been the manual delineation of eye structures, a rather time consuming and error-prone task, to be conducted in multiple MRI sequences simultaneously. This situation, and the lack of tools for accurate eye MRI analysis, reduces the interest in MRI beyond the qualitative evaluation of the optic nerve invasion and the confirmation of recurrent malignancies below calcified tumors. In this manuscript, we propose a new framework for the automatic segmentation of eye structures and ocular tumors in multi-sequence MRI. Our key contribution is the introduction of a pathological eye model from which Eye Patient-Specific Features (EPSF) can be computed. These features combine intensity and shape information of pathological tissue while embedded in healthy structures of the eye. We assess our work on a dataset of pathological patient eyes by computing the Dice Similarity Coefficient (DSC) of the sclera, the cornea, the vitreous humor, the lens and the tumor. In addition, we quantitatively show the superior performance of our pathological eye model as compared to the segmentation obtained by using a healthy model (over 4% DSC) and demonstrate the relevance of our EPSF, which improve the final segmentation regardless of the classifier employed.

## Introduction

Common forms of ocular cancer are related to high morbidity and mortality rates [1]. Imaging of these tumors has generally been performed using 2D Fundus imaging, 2D US or 3D Computed Tomography (CT). Recently however, Magnetic Resonance Imaging (MRI) has gained increased interest within the ophthalmic community, mainly due to its remarkable soft tissue intensity contrast, comparable spatial resolution capabilities to 3D CT and non-ionizing properties [2]. Concretely, MRI is becoming a key modality for pre-treatment diagnostics of tumor extent, especially for retinoblastoma in children, and is gaining a great interest for treatment planning with external beam radiotherapy of uveal melanomas in adults. Examples of these are the works by Beenakker et al. [3] that imaged uveal melanoma at 7-Tesla high-resolution or more recently, measurement comparisons between US and MRI, for assessing tumor dimensions [4].

**Fig 1:**
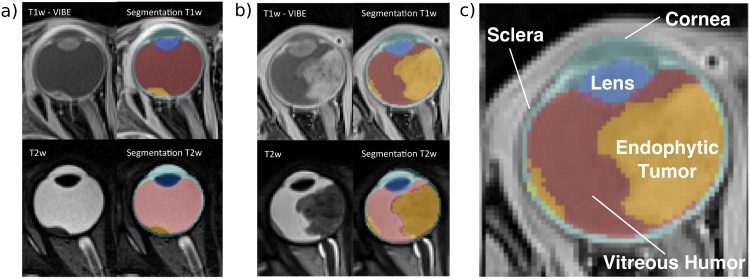
Patients with retinoblastoma. (a)-(b) Two example patients imaged with 3D T1-Weighted (T1w) VIBE & T2-Weighted (T2w) MRI (c) The eye lens (dark blue), vitreous humor (red) and sclera / cornea (light blue) are highlighted. Endophytic tumors delineated in yellow. All annotated regions were delineated manually by an expert radiologist.

For the case of retinoblastoma, a tumor that most often takes root and develops from the retina into the vitreous humor of children eyes [2] (Fig 1), MRI is required to observe possible tumor invasion within the optic nerve, or to evaluate the appearance of recurrent tumors after treatment. Occasionally, treated tumors present second recurrent malignancies under the calcified area, a pathology that can more easily be observed via MRI [1]. In this context, having accurate 3D segmentations of eyes with pathology would help better characterize and quantify intraocular tumors more effectively. This would not only allow for reliable large-scale longitudinal treatment-response studies but would also allow for direct imaging and targeting of tumors during treatment procedures, such as the applied in brachytherapy/cryotherapy to children with retinoblastoma [5].

Unfortunately, and in contrast to neuro(-brain)imaging [6–11], the use of computer-aided techniques for 3D segmentation of the eye remains limited in ocular MRI and, to the best of our knowledge, none address the eye tumor segmentation problem. The accuracy of the existing brain tumor techniques strongly relies on many imaging sequences and on the tumor type they are tailored to (*e.g.* glioma or meningioma,). Thus, their direct application to ocular tumors is not straightforward, considering that only a few contrast images are usually available and, most importantly, they appear as extremely small structures as compared to brain tumors (Fig 1a)).

Today, existing methods for ocular segmentation of healthy structures in Magnetic Resonance (MR) (and CT) rely on semi-automatic techniques based on parametrical models dedicated to segment the eye regions [12], thus only allowing for a coarse segmentation of different eye parts (*e.g.* eye lens or Vitreous Humor (VH)). Also, other approaches such as the one presented by Beenakker et al. [13] aimed at measuring the three-dimensional shape of the retina to study abnormal shape changes and peripheral vision. Along this lines, Active Shape Models (ASMs), originally introduced by Cootes et al. [14] have recently been proposed by our group to more precisely delineate eye anatomy using T1w Volumetric Interpolated Brain Examination (VIBE) MR and CT images [15, 16]. In that work, the main goal was to delineate healthy eye structures, but the challenge of eye tumor delineation has yet to be addressed.

### Contributions of this work

In this paper we present a novel segmentation framework dedicated to ocular anatomy. We present a set of eye delineation techniques in 3D MRI, both for healthy structures and for pathological tissue. In a nutshell, our contributions can be summarized as follows:

- We present a new Pathological Model (PM) of the eye, built out of pathological patient eyes and compare the results with the Healthy Model (HM) presented in [15], achieving better healthy tissue segmentation performance.
- We introduce novel EPSF, derived from our pathological Active Shape Model (ASM), which help characterize pathological tissue, even when only small amounts of training data is available.
- We introduce a novel automatic segmentation framework tailored to ocular tumors. Similar to top ranking algorithms for brain tumor segmentation [17], our method makes use of a Markov Random Field (MRF) to represent the presence of healthy and pathological tissue, allowing for both local and neighborhood information to be utilized in a joint manner. Unlike existing brain tumor techniques however, we encode prior information of the tumors by means of an active shape pathological model, contrasting the use of typical brain atlas priors or existing ASMs of healthy eyes.
- Furthermore, we validate our framework on a clinical dataset of T1w VIBE and T2w MRI from retinoblastoma patients and show that when EPSF are used, performance differences between state-of-the-art deep networks and other simpler classifiers such as Random Forests (RF) are minor and improvements are visible across all cases.

The remainder of this manuscript is divided in the following sections. First, materials and methods section present the clinical dataset, the segmentation of the healthy structures, the eye feature extraction process and our mathematical framework. Second, results comparing the classification performance between different experiments for both healthy tissue segmentation and pathological eye tissue delineation are presented. Third, we discuss about current eye treatment strategies and show the contributions of the presented approach for the future of ocular tumors in the MRI.

## Materials and methods

**Fig 2:**
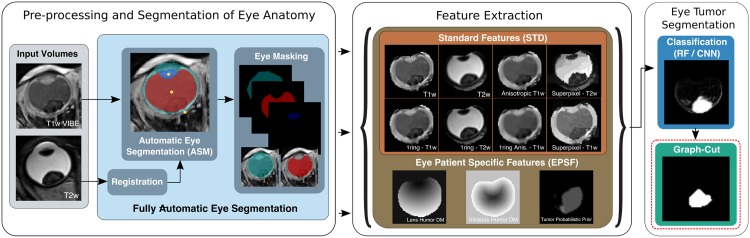
Proposed framework for automatic whole eye segmentation. T1w and T2w 3D volumes are combined with EPSF features. These features are used to train a RF / Convolutional Neural Network (CNN) classifier, serving as the data term in Graph-Cut optimization.

Our framework first makes use of an ASM [14] trained on patient data that contains tumors and which we refer to from now on as *Pathological Model (PM)*. Note that this model is trained exclusively on pathological eye MRI data, in contrast to our previous work [15, 16]. This model aims at delineating the regions of the sclera, the cornea, the lens and the vitreous humor in an automatic fashion. From this pathologically-based ASM, we propose the use of EPSF to characterize pathological tissue within healthy anatomy. We then leverage these features in a classical Markov Random Field (MRF) model to accurately segment eye tumors. Fig 2 illustrates the complete framework which we now describe in detail.

### Dataset

Our dataset is composed of 16 children eyes with endophytic retinoblastoma. The volumes represent a section of the head of the patient, both eyes and the optic nerve. MRI was performed in a 3T Siemens Verio (Siemens, Erlangen, Germany), with a surface head coil of 32 channels. Acquisition was done with patients under general anesthesia. The images are gadolinium enhanced T1w Gradient Echo (GE) VIBE (repetition time/echo time, 19/3.91 ms; flip angle, 12°; Field-of-View, 175 × 175 *mm*^2^; Number of averages, 1, GRAPPA, acceleration factor 2, 3D acquisition, 120 slices, 12:51 acquisition time) acquired at two different spatial resolutions (0.416 × 0.416 × 0.399 mm and 0.480 × 0.480 × 0.499 mm, respectively) and T2w Spin Echo (SE) (repetition time/echo time, 1000/131 ms; flip angle, 120°; Field-of-View, 140 × 140 *mm*^2^ mm; Number of averages, 3.7, 3D acquisition, 60 slices, 8:21 acquisition time) with a resolution of 0.453 × 0.453 × 0.459 mm (See S1 and S2 Files for more details about the sequences parameters). All patient information in our study was anonymized and de-identified by physicians prior to our analysis, and the study was approved by the Cantonal Research Ethics Committee of Vaud. For each eye, the sclera, the cornea, the vitreous humor, the lens and the tumor were manually segmented by an expert radiologist (see Fig 1). We use these manual segmentations as ground truth for quantitative comparisons.

### Pre-processing and segmentation of eye anatomy

Starting from the head-section patient MRIs containing ocular tumors, we begin by automatically locating the Region of Interest (ROI) around each eye. Following the landmark-based registration described in [15, 18], we detect the center of the VH, the center of the lens and the optic disc and use this information to align the eyes to a common coordinate system. This pre-processing allows us to form a coherent dataset representing the same anatomical regions across subjects. In particular, we let our image data *X* for a patient *n* consist of both T1w VIBE and T2w volumes which have been co-registered using a rigid registration scheme, rescaled to a common image resolution, intensity-normalized for all eye volumes [19] and cropped to the ROI. The intensity histogram equalization presented in [19] will help preserving choroid and VH boundaries albeit possible MRI scanner interferences due to coil-related effects.

ASM are powerful tools that enable the encoding of shape and intensity information across a dataset of patients, allowing for the variability of the data to be characterized. For the case of eye structure ASMs, this is achieved by encoding all landmarks of interest into a common shape model which will learn the most relevant deformations within the dataset. Using dimensionality reduction tools, such as Principal Component Analysis (PCA) or Independent Component Analysis (ICA), we can reduce the dimensionality of the model and rely on common trends rather than on specific patient details. In order to build our ASM we rely on the work presented in [20] and later on adapted in [16] and [15], starting with an atlas creation phase, followed by an extraction of a point-based shape variation model representing the structural deformation within the population. Extraction of the intensity profiles perpendicular to the surfaces at every specific landmark is then performed.

**Fig 3:**
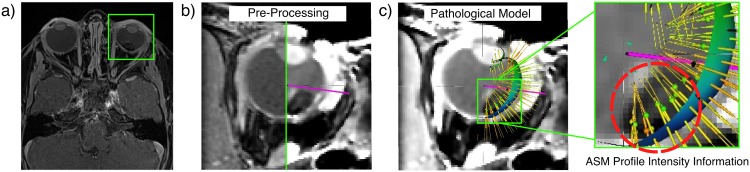
Learning a Pathological Eye Model (PM). We follow the steps in [15] to (a) automatically detect the eye in the 3D MRI, followed by a set of b) image pre-processing techniques to learn information of pathological and healthy structures jointly using c) intensity profiles containing pathological information.

Using manual delineations of the sclera and the cornea, the eye lens and the VH we learn an ASM [14] for these structures jointly. Note that we do not include the delineations of tumors inside the model, but implicitly encode this information from the profile intensity information for the sclera and the VH, as can be seen in Fig 3. With this, we can segment such healthy structures in any subsequent eye MRI. As we will show in Sec. *Results*, learning an ASM on pathological patient data provides improved segmentation accuracy as compared to healthy-patient ASM models eye models [15].

Furthermore, in order to ease the process of learning the tumor classifier, we will only consider voxels at a Euclidean distance of *θ* = (0, 2) mm from the VH delineation result. That is, we only evaluate voxels that are close and within the VH, upper bounded by the maximum segmentation error of the ASM (see Fig 2, brown-colored box). The main purpose at this stage is to focus exclusively on the tumor segmentation, reducing the observable region to the VH plus an additional confidence at a distance *θ* from the boundary.

### Feature extraction

We let $f_{i}^{STD}$ at voxel location *i* denote Standard Features (STD) defined as the concatenation of T1w and T2w voxel intensities, the anisotropic T1-weighted (A-T1w), and the average 6-voxel von-Neumann neighbors (6 nearest neigbors) of the T1w, T2w and A-T1w. On their own such features provide standard intensity information to the appearance of both healthy and pathological tissue (see Fig 2, brown-colored box).

Given a segmentation of healthy eye structures, we are interested in establishing image features that provide appearance and *contextual* information regarding pathological tissue as well. To achieve this goal, we begin by applying the learned ASM (see Sec. *Pre-processing*) to the eye MRI, thus providing a delineation boundary for the lens and VH. Then, we assign to each voxel *i* in the volume a rank relative to the Euclidean distance to the surface border of both the lens and the VH. More specifically, the rank at each voxel is defined as,

$$\begin{array}{c} f_{i}^{l}=\min_{b\in B_{l}}{\left|i-b\right|}_{2}^{2},\;\text{and}\;f_{i}^{vh}=\min_{b\in B_{vh}}{\left|i-b\right|}_{2}^{2}, \end{array}$$ (1)

where *B*_*l*_ and *B*_*vh*_ are the boundary voxels of the eye lens and VH, respectively. These features can be interpreted as distance maps resulting from the pathological ASM segmentation.

In addition, in order to leverage prior information on the tumor locations, we also use the ground truth tumor locations within our training set, to construct a tumor location likelihood feature, $f_{i}^{t}=\frac{1}{N}\sum_{n_{1}}^{N}Y_{i}$, where *Y*_*i*_ = 1 if the voxel at location *i* is part of the tumor and where *N* is the total number of volumes in the training set. Smoothing **f**^*t*^ is then performed by using a 3D gaussian kernel (*σ* = 3 mm) to regularize the tumor prior. This value was arbitrarily chosen as to create a smooth yet precise representation of the tumor distribution inside the eye. In Fig 2 you can find an example of these feature maps.

Our EPSF for each voxel is then constructed by concatenating the following rank-values: $f_{i}^{EPSF}=\left[f_{i}^{l},f_{i}^{vh},f_{i}^{t}\right]$. As illustrated in Fig 2, this effectively characterizes patient-specific anatomy (via $f_{i}^{l}$, $f_{i}^{vh}$) and a location specific tumor likelihood (via $f_{i}^{t}$), allowing both local and global information to be encoded in a compact feature at voxel level.

### Eye tumor segmentation

#### General mathematical framework

Now, to segment the eye tumor tissue, we make use of a MRF to model the joint distribution of all the voxels P(Y,X|M), where *Y* = ∪_*i*_*Y*_*i*_, *Y*_*i*_ ∈ {0, 1} represents the presence of a tumor at location *i*, *X* is the MR volume and $M=M_{1},\ldots ,M_{S}$, represents the different healthy eye structures,

$$\begin{array}{c} P\left(Y,X|M\right)=\frac{1}{Z}\prod_{i}P\left(X_{i}|Y_{i},M\right)\prod_{j\in N_{i}}P\left(Y_{i},Y_{j}|M\right), \end{array}$$ (2)

with normalization factor *Z*, likelihood model $P(X_{i}|Y_{i},M)$ and smoothness prior $P(Y_{i},Y_{j}|M)$. From this, we can simplify this expression to,

$$\begin{array}{c} P\left(Y,X|M\right)=\frac{1}{Z}\prod_{i}P\left(f_{i}|Y_{i}\right)\prod_{j\in N_{i}}P\left(Y_{i},Y_{j}\right), \end{array}$$ (3)

by assuming that (1) the prior is independent of the underlying eye structure and that a single model accurately describes the neighborhood interactions and (2) instead of modeling the likelihoods as a function of a given structure, we approximate it by leveraging our patient-specific features that are implicitly indexed on M. Using this model, we can then use a standard Graph-cut optimization [21, 22] to infer the tumor segmentation. The presented approach is particularly powerful because the topology of our segmentation is not restricted to a single structure and allows refining segmentation on multiple isolated parts.

#### Likelihood prior

The likelihood prior, defined as *P*(**f**_*i*_|*Y*_*i*_), provides the probability for every voxel *i* within the 3D volume to represent either tumor or healthy tissue *Y*_*i*_ = {0, 1}. In this work we conduct a set of experiments with two scenarios, a) a rather simplistic voxel-based RF classifier and b) a state-of-the-art CNN [11], which leverages the power of 3D convolutions to perform the same classification task.

**RF classification:** We train a RF classifier [23] with 200 trees, using all positive voxels (*Y*_*i*_ = 1) in the training set and 20% of the negative voxels (*Y*_*i*_ = 0) to balance the number of samples. As in [24], SLIC superpixels [25] are computed on each 2D-MR slice (*i.e.* region size of 10 voxels and a regularization factor *r* = 0.1), from which mean superpixel intensity at position *i* was aggregated for both T1w and T2w. SLIC features support the voxel-wise classification by providing intensity context based on the surrounding area.

The number of trees was selected by reaching convergence with Out-of-Bag estimation, testing RF performance with a varying number of trees from 50 to 1000 and choosing the minimum number of trees to reach convergence.

**CNN classification:** We train a modified version of the 3D CNN presented in [11], known as *DeepMedic*. The model we employ consists of 8 convolutional layers followed by a classification layer. All hidden layers use 3^3^-sized kernels, which leads to a model with a 17^3^-sized receptive field. In this task, where the ROI is smaller than for brain cases (80*x*80*x*83 voxels), processing around each voxel is deemed enough for its classification and thus, the multi-resolution approach of the original work is not used. We reduced the number of Feature Maps (FM) in comparison to the original model {15, 15, 20, 20, 25, 25, 30, 30} at each hidden layer respectively, not only to mitigate the risk of overfitting given the small dataset, but also to the reduce the computation burden.

To enhance the generalization of the CNN, we augment the training data with reflections over the x, y and z axis. We use *dropout* [26] with 50% rate at the final layer, which counters overfitting by disallowing feature co-adaption. Finally, we utilize *Batch normalization* [27], which accelerates training’s convergence and also relates the activation of neurons across a whole batch, regularizing the model’s learnt representation. The rest of *DeepMedic* parameters were kept similar to the original configuration [11], as preliminary experimentation showed satisfactory behavior of the system.

#### Smoothness prior and refinement

Following the mathematical model in Eq (3), we are interested in having a smoothness prior that favors pairs of labels that are deemed *similar* to one another. In our case, we estimate the similarity intensity values from both T1w and T2w with a parametric model of the form

$$\begin{array}{c} P\left(Y_{i},Y_{j}\right)=\alpha \cdot e^{-\frac{1}{2\sigma_{T_{1}}^{2}}{\left(T_{1}^{i}-T_{1}^{j}\right)}^{2}}+\left(1-\alpha \right)\cdot e^{-\frac{1}{2\sigma_{T_{2}}^{2}}{\left(T_{2}^{i}-T_{2}^{j}\right)}^{2}}, \end{array}$$ (4)

where $T_{1}^{i}$ is the intensity value at voxel *i* of the T1w VIBE volume (and similarly for $T_{2}^{i}$), *α* ∈ (0, 1) is a bias term between T1w and T2w importance and where ($\sigma_{T_{1}}^{2},\sigma_{T_{2}}^{2}$) are the voxel intensity variances of tumor locations in *T*_1_ and *T*_2_.

## Results

### Contribution of Pathological Eye Model

We performed a leave-one-out cross validation experiment on the presented *Pathological Model* (PM). We compare its accuracy with our previous work [15], a Healthy Model (HM) constructed of 24 healthy eyes. We furthermore tested the performance of combining both the HM and the PM into a Combined Model (CM) built out of 40 patient eyes (24 healthy and 16 pathological). Table 1 reports the DSC accuracy, which measures the volume overlap between our results and the Ground Truth (GT), for the three different models (*i.e.* PM, HM and CM) when segmenting healthy tissue on our pathological patient dataset. We observe that the PM outperforms the existing HM on average by a ≈4.5% and the CM by a ≈3.5% (statistical significance evaluated using a paired t-test showing a *p* < 0.05 for both cases). Extended results in S1 Table.

**Table 1:** Eye anatomy DSC: Our Pathological Model (PM) shows more accurate results than the Healthy Model (HM) from [15] and the Combined Model (CM), especially for the region of the lens. (*) *p* < 0.05. The table indicates both the Dice Similarity Coefficient (DSC) and the maximum surface segmentation error or *Hausdorff Distance (HD)*, in mm.

DSC [%]	**Sclera**	**Vitreous Humor**	**Lens**	**Average**
PM (%)	**94.62 ± 1.9**	**94.52 ± 2.36**	**85.67 ± 4.68**	91.51 ± 1.49*
HM—[15] (%)	92.27 ± 4.13	91.62 ± 4.42	76.97 ± 20.28	86.95 ± 9.24
CM (%)	92.45 ± 2.99	91.85 ± 3.07	79.22 ± 14.08	87.84 ± 6.38
Distance [mm]	**Sclera**	**Vitreous Humor**	**Lens**	**Average**
PM-Hausdorff	1.30 ± 0.29	1.35 ± 0.28	0.70 ± 0.22	N.A.
PM Avg.Error	0.46 ± 0.16	0.46 ± 0.15	0.28 ± 0.06	0.401 ± 0.124

### Contribution of EPSF for tumor segmentation

**Fig 4:**
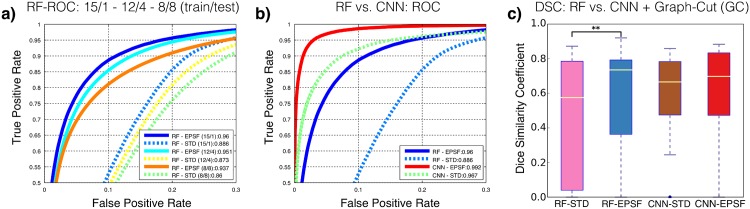
Classification performance. (a) ROC curve depicting the effect of varying amounts of (training/test) data for RF classification with and without EPSF. (b) ROC curve for both experiments (CNN / RF) with STD and with EPSF. (c) DSC tumor segmentation results for STD vs. EPSF. The latter shows better results for both cases (** = *p* < 0.01).

We evaluated the tumor segmentation performances of our strategy when using a RF and a CNN for the MRF likelihood model. In both cases, we tested the performance of **f**^**STD**^ features only and combined **f**^**STD**,**EPSF**^ features as well. For each scenario we optimized *θ* using cross validation, giving values of *θ* = 0 mm for RF-STD and *θ* = 2 mm otherwise. Fig 4(a) indicates the relevant contribution of EPSF, even when trained on very small amounts of data for the RF classifier. Here we see EPSF provides more robustness towards improving the classifier’s ability to generalize when trained with limited amounts of data. Fig 4(b) illustrates the ROC performance of the likelihood models and the different feature combinations. In particular, we see that regardless of the classifier used, EPSF provide added performance in the likelihood model. Similarly, improved segmentation performances are attained with EPSF once inference of the MRF is performed, as depicted in Fig 4(c). Optimal parameters for the smoothness term are *α* = 0.3, *λ* = 0.7 for RF and *α* = 0.4, *λ* = 0.5 for CNN. Table 2 illustrates this point more precisely by showing the average DSC and Hausdorff Distance (HD) scores before and after MRF inference is performed and with different combinations of classifiers and features.

**Table 2:** DSC performance for different scenarios before and after Graph-cut (GC) inference. Hausdorff Distance (HD) and Mean Distance Error after GC inference. Complete results in S1 Table. Experiments were computed on ⋄: Macbook Pro Intel-Core™ i7 16GB—2, 5 GHZ & †: Intel-Core™ i7 6700 32GB with Nvidia GTX Titan X^®^.

	RF + EPSF	RF + STD	CNN + EPSF	CNN+STD
DSC [%]	36.16 ± 27.85	24.49 ± 22.25	56.87 ± 29.38	53.67 ± 24.29
DSC (GC) [%]	**58.50 ± 32.07**	46.15 ± 34.88	**62.25 ± 26.27**	57.64 ± 28.35
HD (GC) [mm]	**3.998 ± 4.130**	7.580 ± 8.776	**0.977 ± 0.729**	3.125 ± 3.971
Mean Error [mm]	**0.641 ± 0.884**	3.414 ± 3.874	**0.175 ± 0.062**	0.266 ± 0.172
Training time [min]	**2.36** ⋄	2.21 ⋄	**150** †	150 †

**Fig 5:**
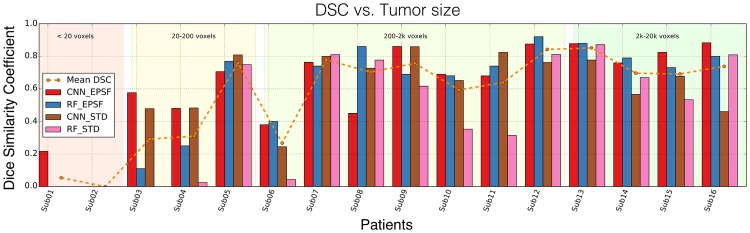
DSC vs. Tumor size. Average results for different combination of classifiers and feature sets. EPSF improves overall classification results over STD features consistently.

**Fig 6:**
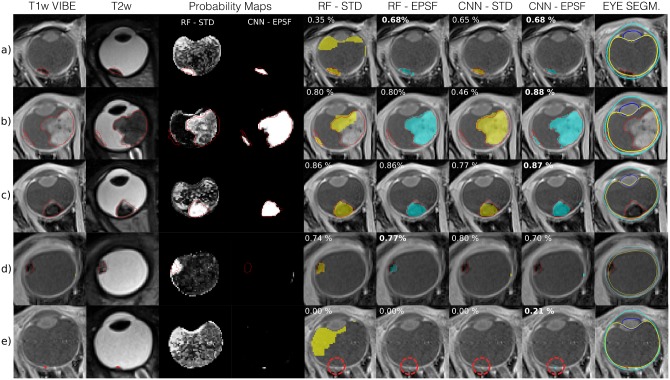
Example segmentation results. the tumor ground truth is delineated in red. Probability Maps, *P*(**f**_*i*_|*Y*_*i*_), for worst (RF-STD) and best (CNN-EPSF) scenarios. Final column shows the pathological model (PM) eye segmentation results.

In Fig 5, we show the DSC performance of the different features and classifiers as a function of tumor size for each patient in the dataset. Note that despite ocular tumors being smaller than brain tumors, DSC values are in line with those obtained for brain tumor segmentation tasks [17]. This illustrates the good performance of our strategy even though DSC is negatively biased for small structures. Except for the two smallest tumors in our dataset (<20 voxels), DSC scores attained are good, with a HD under the MRI volume’s resolution threshold for CNNs. Our quantitative assessment is supported by the visual inspection of the results (see Fig 6).

## Discussion

This work introduces a multi-sequence MRI-based eye and tumor segmentation framework. To the best of our knowledge, this is the first attempt towards performing qualitative eye tumor delineation in 3D MRI. We have presented a PM of the eye that encodes information of tumor occurrence as part of the ASM for various eye regions. Introducing pathological information results in a more robust model (with a significantly lower standard deviation), able to improve segmentation for the regions of the sclera, the cornea, the vitreous humor and the lens. The delineation is followed by a binary masking *θ*, where the complexity of the tumor segmentation problem is reduced to the eyeball region, and is upper bounded by the maximum euclidean distance error during segmentation. Despite the simplicity of the masking, the gain in performance is significant for all the tested classifiers.

From a clinical perspective, we have presented a new tool for a) measuring the eye tumor size, a task which is now only performed qualitatively in 3D MRI, b) ocular tumor follow-ups, by providing a qualitative estimation of cancer volume variations to enable tracking of the effectiveness of treatment (*e.g* tumor shrinking over time, …) as well as c) a new approach to study cancer recurrence. Note that recurrence appears often for calcified retinoblastoma tumors [2], developing under the visible area a clinician would observe during Fundus and US, thus making MRI and up to a certain extent, US, the sole option to detect recurrent tumors under calcified tissue inside the eye.

Indeed, MRI and US remain the most effectives ways to discover second recurrent malignancies appearing behind the calcified ocular tumors. During first explorations, one of the most important goals for doctors using MRI is to evaluate the totality of the tumor extent and whether there exists any invasion outside the ocular cavity or towards the optic nerve. In this direction, the evaluation of relatively small tumors, such as the ones presented in this paper (e.g. smaller than 20 voxels), are mostly conducted with alternative image modalities such as Fundus Image Photography and despite that fact that the tumor segmentation achieved was not ideal, it provided a glimpse of the potential for using MRI to infer the size of such tumor. In its current form, this technique would be mainly focused on estimating the extent of tumors whose size exceeds 50 voxels and beyond in area (>3mm diameter) as well as whenever alternative exploration techniques, such as Fundus or Ultrasound, cannot rule out the presence of tumors in the region of interest.

Here, we used two sequences for imaging the eyes (T1w VIBE and T2w) which were specifically selected in collaborations with expert radiologist from our clinical institution. Their choice was based on the protocol suggested in the European Retinoblastoma Imaging Collaboration (ERIC) guidelines for retinoblastoma imaging [2, 28]. The clinician’s feedback specified that the spatial resolution and intensity contrast offered by these 2 sequences provided resolute information about the nature of the tumors and calcifications. At the same time, the use of Diffusion-Weighted (DW) MR imaging was thought to drive to less complete clinical evaluations and decisions [2, 28, 29].

Moreover, when it comes to evaluating the quality of the segmentation, there is a remarkable difference between the delineation of small (<20 voxels) and big (>2*k* voxels) pathologies. Compact retinoblastomas can be up to four orders of magnitude smaller than such cases. This variation poses a challenge for the presented framework and highly influences the final volume overlap, measured in the form of DSC. A clear example of this challenging segmentation is the one we can observe in Fig 6e), where the small size of the tumor makes segmentation with our approach unreliable. In the supplementary material we show five additional examples of the presented results (see S1 Fig). To provide a more reasonable measure of the quality of the delineation, we opted for the Hausdorff Distance (HD), a widely spread method in medical imaging to measure segmentation based on distance to the surface GT. Having a look at both DSC and HD, we clearly notice that despite the DSC of the RF with EPSF being larger than the CNN with STD features, the latter offers a more robust segmentation (*supplementary material includes extended results for DSC*). A limitation of the presented CNN results, though, was to be able to segment tumors similar to the one in Fig 6d) (close to the lens), an issue that would potentially be resolved by increasing the number of training samples. In the future, the presented work should be evaluated with a larger dataset where more samples with tumors from varying sizes are investigated. Also, tumors with similar imaging conditions, such as uveal melanoma, would be good candidates for performing the evaluation. Furthermore, and in order to potentiate the use of the presented tool, we will offer a functional copy of the pipeline alongside a minimal dataset for segmenting ocular tumors in the eye in 3D MRI online (*Available at* http://www.unil.ch/mial/home/menuguid/software.html).

One of the most important constraints of current eye MR imaging in ophthalmology are the limitation in terms of resolution, the scanning time, and the difficulty to disentangle small tumors from the choroid, towards both the inside (endophytic) and the outside of the eyes (exopythic). To compensate for this, multiple image modalities (*e.g.* US, CT, Fundus) are evaluated in order to decide about the best treatment strategy. Among the current challenges to improve this decisive step, one of the most relevant is to find a way to connect multiple image modalities in a robust manner. That is, connecting image modalities at different scales (MRI, Fundus, Optical Coherence Tomography (OCT) or US) and use common anatomical landmarks to validate the multi-modal fusion. This contribution would not only imply refining the quality of the delineation and treatment based on MRI (such as the framework presented in this manuscript) and other medical image sequences, but it would also support clinicians during the process of decision making, evaluation of tumor extent and patient follow-up, enabling co-working clinical specialists from various backgrounds and modality preferences into the same common perspective.

## Conclusion

We introduced a framework for multi-sequence whole eye segmentation and tumor delineation in MRI. We leveraged the use of pathological priors by means of an ASM and introduced a new set of features (EPSF) that characterize shape, position and tumor likelihood. When combined with traditional features, EPSF are robust and effective at segmenting pathological tissue, particularly when tumors are small. Our approach shows comparable DSC and HD performances to state-of-the-art brain tumor segmentation even when limited amounts of data are available. Both RF and CNN show improved results with our features, while the former performs almost as equally as the latter and is trained in a fraction of the time. One future direction we plan to investigate is how our approach can be extended and modified in order to automatically segment both healthy and pathological tissue structures in adult uveal melanoma patients.

## Supporting information

S1 File T1w Gradient Echo (GE) VIBE Sequence. Detailed sequence information for reproducing the MRI acquisition.
(PDF)

S2 File T2w Spin Echo (SE) Sequence. Detailed sequence information for reproducing the MRI acquisition.
(PDF)

S1 Table Complementary results. Complete results comparing HM vs. PM vs. CM. Additional details about the classificacion performance for all 4 experiments (RF-STD, RF-EPSF, CNN-EPSF and CNN-STD) and for all patients.
(XLSX)

S1 Fig Automatic segmentation of eye structures and tumors. Five additional examples for the segmentation performed on retinoblastoma patients. a-b) Two examples including retinal detachment. c) Small tumor located close to the optic nerve. d) Smallest tumor in the dataset (≈20 voxels). T2w sequence does not show any sign of tumor. e) Mid-sized tumor close to the optic nerve.
(TIFF)
