# Supplementary material for: Multi-channel MRI segmentation of eye structures and tumors using patient-specific features
Source: PLoS One. 2017 Mar 28;12(3):e0173900. doi: 10.1371/journal.pone.0173900 (PMC5369682; doi:10.1371/journal.pone.0173900)
Supplement: S1 File — Detailed sequence information for reproducing the MRI acquisition. (PDF) [file pone.0173900.s001.pdf]

**\\SkyraFit\Skf_Neuro\B Orbites\RetinoSousAg\t1_vibe_fs_tra_UHR_0.3_32cx_Retino**
TA: 12:51 PM: FIX Voxel size: 0.3×0.3×0.3 mmPAT: 2 Rel. SNR: 1.00 : fl

Properties

Prio recon	Off
Load images to viewer	On
Inline movie	Off
Auto store images	On
Load images to stamp segments	Off
Load images to graphic segments	Off
Auto open inline display	Off
Auto close inline display	Off
Start measurement without further preparation	Off
Wait for user to start	Off
Start measurements	Single measurement

Routine

Slab group	1
Slabs	1
Dist. factor	20 %
Position	L0.9 P2.2 H2.2 mm
Orientation	T > C1.7 > S1.3
Phase enc. dir.	R >> L
AutoAlign	Head > Brain
Phase oversampling	5 %
Slice oversampling	46.7 %
Slices per slab	120
FoV read	175 mm
FoV phase	93.8 %
Slice thickness	0.3 mm
TR	19.00 ms
TE	4.11 ms
Averages	1
Concatenations	1
Filter	Prescan Normalize, Elliptical filter
Coil elements	HEA;HEP

Contrast - Common

TR	19.00 ms
TE	4.11 ms
Flip angle	12.0 deg
Fat suppr.	Q-fat sat.
Lines Per Shot	132
Water suppr.	None
Dixon	Off

Contrast - Dynamic

Averages	1
Averaging mode	Short term
Reconstruction	Magnitude
Measurements	1
Multiple series	Each measurement

Resolution - Common

FoV read	175 mm
FoV phase	93.8 %
Slice thickness	0.3 mm
Base resolution	576
Phase resolution	100 %
Slice resolution	75 %
Phase partial Fourier	Off
Slice partial Fourier	Off

Resolution - Common

Trajectory	Cartesian
View sharing	Off
Interpolation	Off

Resolution - iPAT

PAT mode	GRAPPA
Accel. factor PE	2
Ref. lines PE	32
Accel. factor 3D	1
Reference scan mode	Integrated

Resolution - Filter Image

Image Filter	Off
Distortion Corr.	Off
Prescan Normalize	On
Unfiltered images	Off
Normalize	Off
B1 filter	Off

Resolution - Filter Rawdata

Raw filter	Off
Elliptical filter	On
POCS	Off

Geometry - Common

Slab group	1
Slabs	1
Dist. factor	20 %
Position	L0.9 P2.2 H2.2 mm
Orientation	T > C1.7 > S1.3
Phase enc. dir.	R >> L
Slice oversampling	46.7 %
Slices per slab	120
FoV read	175 mm
FoV phase	93.8 %
Slice thickness	0.3 mm
TR	19.00 ms
Multi-slice mode	Sequential
Series	Ascending
Concatenations	1

Geometry - AutoAlign

Slab group	1
Position	L0.9 P2.2 H2.2 mm
Orientation	T > C1.7 > S1.3
Phase enc. dir.	R >> L
AutoAlign	Head > Brain
Initial Position	R0.3 A0.1 F28.7
R	0.3 mm
A	0.1 mm
F	28.7 mm
Initial Rotation	89.98 deg
Initial Orientation	Transversal

Geometry - Saturation

Fat suppr.	Q-fat sat.
Water suppr.	None
Dixon	Off
Special sat.	None

Geometry - Tim Planning Suite

Set-n-Go Protocol	Off
Table position	H
Table position	0 mm
Inline Composing	Off

System - Miscellaneous

Positioning mode	FIX
Table position	H
Table position	0 mm
MSMA	S - C - T
Sagittal	R >> L
Coronal	A >> P
Transversal	F >> H
Coil Combine Mode	Adaptive Combine
Save uncombined	Off
Matrix Optimization	Off
AutoAlign	Head > Brain
Coil Select Mode	Off - AutoCoilSelect

System - Adjustments

B0 Shim mode	Standard
B1 Shim mode	TrueForm
Adjust with body coil	Off
Confirm freq. adjustment	Off
Assume Dominant Fat	Off
Assume Silicone	Off
Adjustment Tolerance	Auto

System - Adjust Volume

Position	L0.9 P2.2 H2.2 mm
Orientation	T > C1.7 > S1.3
Rotation	86.20 deg
R >> L	165 mm
A >> P	175 mm
F >> H	36 mm
Reset	Off

System - Tx/Rx

Frequency 1H	123.257577 MHz
Correction factor	1
Gain	High
Img. Scale Cor.	1.000
Reset	Off
? Ref. amplitude 1H	0.000 V

Physio - PACE

Resp. control	Off
Concatenations	1

Inline - Common

View sharing	Off
Flip angle	12.0 deg
Measurements	1
Burn time-to-center	Off
Temporal interpolation	1
3D centric reordering	Off
Time to center	383.7 s

Inline - Inline

Subtract	Off
Measurements	1
StdDev	Off
Liver registration	Off

Inline - Inline

Save original images	On

Inline - MIP

MIP-Sag	Off
MIP-Cor	Off
MIP-Tra	Off
MIP-Time	Off
Save original images	On

Inline - Soft Tissue

Wash - In	Off
Wash - Out	Off
TTP	Off
PEI	Off
MIP - time	Off
Measurements	1

Inline - Composing

Inline Composing	Off
Distortion Corr.	Off

Inline - MapIt

Save original images	On
MapIt	None
Flip angle	12.0 deg
Measurements	1
Contrasts	1
TR	19.00 ms
TE	4.11 ms

Sequence - Part 1

Introduction	On
Dimension	3D
Elliptical scanning	Off
Reordering	Centric
Asymmetric echo	Weak
Contrasts	1
Optimization	Min. TE
Multi-slice mode	Sequential
Bandwidth	140 Hz/Px

Sequence - Part 2

RF pulse type	Normal
Gradient mode	Fast
Excitation	Slab-sel.
RF spoiling	On
Incr. Gradient spoiling	Off

Sequence - Assistant

Mode	Off
Allowed delay	0 s
