# Supplementary material for: Multi-channel MRI segmentation of eye structures and tumors using patient-specific features
Source: PLoS One. 2017 Mar 28;12(3):e0173900. doi: 10.1371/journal.pone.0173900 (PMC5369682; doi:10.1371/journal.pone.0173900)
Supplement: S2 File — Detailed sequence information for reproducing the MRI acquisition. (PDF) [file pone.0173900.s002.pdf]

\\SkyraFit\Skf_Neuro\B Orbites\RetinoSousAg\t2_spc_tra_restore ISO 0.44 HR FOV 140
TA: 8:21 PM: FIX Voxel size: 0.2×0.2×0.5 mmPAT: Off Rel. SNR: 1.00 : spcR

Properties

Prio recon	Off
Load images to viewer	On
Inline movie	Off
Auto store images	On
Load images to stamp segments	Off
Load images to graphic segments	Off
Auto open inline display	Off
Auto close inline display	Off
Start measurement without further preparation	Off
Wait for user to start	Off
Start measurements	Single measurement

Routine

Slab group	1
Slabs	1
Position	R0.3 A16.4 F31.5 mm
Orientation	T > S1.5 > C0.2
Phase enc. dir.	R >> L
AutoAlign	Head > Brain
Phase oversampling	0 %
Slice oversampling	20.0 %
Slices per slab	60
FoV read	145 mm
FoV phase	100.0 %
Slice thickness	0.45 mm
TR	1000 ms
TE	131 ms
Averages	3.7
Concatenations	1
Filter	Raw filter, Prescan Normalize
Coil elements	HEA;HEP

Contrast - Common

TR	1000 ms
TE	131 ms
MTC	Off
Magn. preparation	None
Flip angle	120 deg
Fat suppr.	None
Blood suppr.	Off
Restore magn.	On

Contrast - Dynamic

Averages	3.7
Reconstruction	Magnitude
Measurements	1
Multiple series	Each measurement

Resolution - Common

FoV read	145 mm
FoV phase	100.0 %
Slice thickness	0.45 mm
Base resolution	320
Phase resolution	99 %
Slice resolution	50 %
Phase partial Fourier	Allowed
Slice partial Fourier	Off
Interpolation	On

Resolution - iPAT

PAT mode	None

Resolution - Filter Image

Image Filter	Off
Distortion Corr.	Off
Prescan Normalize	On
Unfiltered images	Off
Normalize	Off
B1 filter	Off

Resolution - Filter Rawdata

Raw filter	On
Elliptical filter	Off

Geometry - Common

Slab group	1
Slabs	1
Position	R0.3 A16.4 F31.5 mm
Orientation	T > S1.5 > C0.2
Phase enc. dir.	R >> L
Slice oversampling	20.0 %
Slices per slab	60
FoV read	145 mm
FoV phase	100.0 %
Slice thickness	0.45 mm
TR	1000 ms
Series	Interleaved
Concatenations	1

Geometry - AutoAlign

Slab group	1
Position	R0.3 A16.4 F31.5 mm
Orientation	T > S1.5 > C0.2
Phase enc. dir.	R >> L
AutoAlign	Head > Brain
Initial Position	R0.3 A16.4 F31.5
R	0.3 mm
A	16.4 mm
F	31.5 mm
Initial Rotation	89.99 deg
Initial Orientation	T > S
T > S	1.5
> C	0.2

Geometry - Saturation

Fat suppr.	None
Restore magn.	On
Special sat.	None

Geometry - Navigator

Geometry - Tim Planning Suite

Set-n-Go Protocol	Off
Table position	H
Table position	0 mm
Inline Composing	Off

System - Miscellaneous

Positioning mode	FIX

System - Miscellaneous

Table position	H
Table position	0 mm
MSMA	S - C - T
Sagittal	R >> L
Coronal	A >> P
Transversal	F >> H
Coil Combine Mode	Adaptive Combine
Save uncombined	Off
Matrix Optimization	Off
AutoAlign	Head > Brain
Coil Select Mode	Default

System - Adjustments

B0 Shim mode	Tune up
B1 Shim mode	TrueForm
Adjust with body coil	Off
Confirm freq. adjustment	Off
Assume Dominant Fat	Off
Assume Silicone	Off
Adjustment Tolerance	Auto

System - Adjust Volume

Position	Isocenter
Orientation	Transversal
Rotation	0.00 deg
A >> P	263 mm
R >> L	350 mm
F >> H	350 mm
Reset	Off

System - Tx/Rx

Frequency 1H	123.257577 MHz
Correction factor	1
Gain	High
Img. Scale Cor.	1.000
Reset	Off
? Ref. amplitude 1H	0.000 V

Physio - Signal1

1st Signal/Mode	None
Trigger delay	0 ms
TR	1000 ms
Concatenations	1

Physio - Cardiac

Magn. preparation	None
Fat suppr.	None
Dark blood	Off
FoV read	145 mm
FoV phase	100.0 %
Phase resolution	99 %

Physio - PACE

Resp. control	Off
Concatenations	1

Inline - Common

Subtract	Off
Measurements	1
StdDev	Off
Save original images	On

Inline - MIP

MIP-Sag	Off
MIP-Cor	Off
MIP-Tra	Off
MIP-Time	Off
Save original images	On

Inline - Composing

Inline Composing	Off
Distortion Corr.	Off

Sequence - Part 1

Introduction	Off
Dimension	3D
Elliptical scanning	Off
Reordering	Linear
Flow comp.	No
Echo spacing	6.99 ms
Adiabatic-mode	Off
Bandwidth	289 Hz/Px

Sequence - Part 2

Echo train duration	419 ms
RF pulse type	Normal
Gradient mode	Normal
Excitation	Slab-sel.
Flip angle mode	Constant
Turbo factor	85

Sequence - Assistant

Allowed delay	30 s
