# Supplementary figures and images for: Multi-channel MRI segmentation of eye structures and tumors using patient-specific features
Source: PLoS One. 2017 Mar 28;12(3):e0173900. doi: 10.1371/journal.pone.0173900 (PMC5369682; doi:10.1371/journal.pone.0173900)

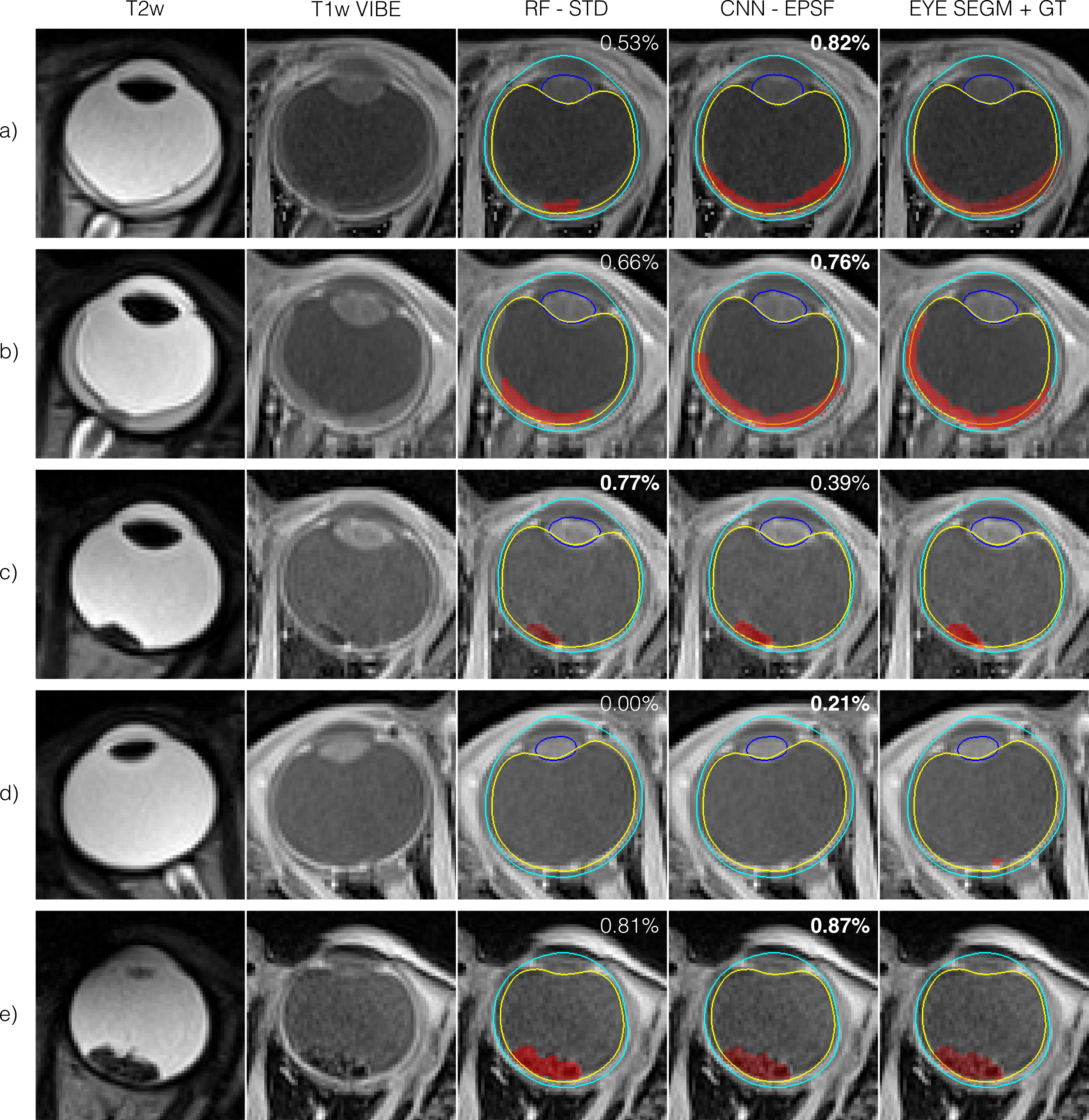

Supplement: S1 Fig — Five additional examples for the segmentation performed on retinoblastoma patients. a-b) Two examples including retinal detachment. c) Small tumor located close to the optic nerve. d) Smallest tumor in the dataset (≈20 voxels). T2w sequence does not show any sign of tumor. e) Mid-sized tumor close to the optic nerve. (TIFF) [file pone.0173900.s004.tiff]
